# Test Platform for Developing New Optical Position Tracking Technology towards Improved Head Motion Correction in Magnetic Resonance Imaging

**DOI:** 10.3390/s24123737

**Published:** 2024-06-08

**Authors:** Marina Silic, Fred Tam, Simon J. Graham

**Affiliations:** 1 Physical Sciences Platform, Sunnybrook Research Institute, Toronto, ON M4N 3M5, Canada; marina.silic@mail.utoronto.ca (M.S.); fred.tam@sri.utoronto.ca (F.T.); 2 Department of Medical Biophysics, University of Toronto, Toronto, ON M5G 1L7, Canada

**Keywords:** magnetic resonance imaging, neuroimaging, motion correction, motion artifacts, optical tracking, head pose estimation, moiré pattern, deep learning, CNN, synthetic dataset

## Abstract

Optical tracking of head pose via fiducial markers has been proven to enable effective correction of motion artifacts in the brain during magnetic resonance imaging but remains difficult to implement in the clinic due to lengthy calibration and set up times. Advances in deep learning for markerless head pose estimation have yet to be applied to this problem because of the sub-millimetre spatial resolution required for motion correction. In the present work, two optical tracking systems are described for the development and training of a neural network: one marker-based system (a testing platform for measuring ground truth head pose) with high tracking fidelity to act as the training labels, and one markerless deep-learning-based system using images of the markerless head as input to the network. The markerless system has the potential to overcome issues of marker occlusion, insufficient rigid attachment of the marker, lengthy calibration times, and unequal performance across degrees of freedom (DOF), all of which hamper the adoption of marker-based solutions in the clinic. Detail is provided on the development of a custom moiré-enhanced fiducial marker for use as ground truth and on the calibration procedure for both optical tracking systems. Additionally, the development of a synthetic head pose dataset is described for the proof of concept and initial pre-training of a simple convolutional neural network. Results indicate that the ground truth system has been sufficiently calibrated and can track head pose with an error of <1 mm and <1°. Tracking data of a healthy, adult participant are shown. Pre-training results show that the average root-mean-squared error across the 6 DOF is 0.13 and 0.36 (mm or degrees) on a head model included and excluded from the training dataset, respectively. Overall, this work indicates excellent feasibility of the deep-learning-based approach and will enable future work in training and testing on a real dataset in the MRI environment.

## 1. Introduction

Many efforts have been made to reduce and eliminate motion artifacts (noise contamination caused by movement) in magnetic resonance imaging (MRI) since the inception of this medical imaging modality. Today, despite the major role MRI plays in healthcare, motion remains a challenging, ongoing problem in the MRI clinic. The typical spatial resolution of anatomical MRI applications at present (~1 × 1 × 1 mm at a magnetic field strength of 3 T) and the timescale of the data collection (seconds to minutes) ensure that many physiological motions can still cause artifacts [1]. The type and severity of motion artifacts are related to various factors, such as the anatomy to be imaged, the MRI signal contrast and spatial encoding prescription, the timing of the motion during the MRI pulse sequence, and the direction and amplitude of motion. This variability necessitates diverse motion correction methods [2]. The “toolbox” of potential solutions is continuously expanding, and it is unlikely that a single solution will ever prevail over the rest [3].

The predominant MRI application is medical visualisation of the brain, in which head movement, subtle motion caused by the respiratory and cardiac cycles, and the effects of muscle relaxation during imaging can all be problematic. A common, useful method of modelling and correcting for these effects has been to assume rigid body motion in three translational and three rotational coordinates, for a total of six degrees of freedom (6 DOF). Under this assumption, numerous retrospective and prospective motion correction methods have been devised that are based on “tracking” (measuring) these DOF during MRI [4]. Retrospective correction involves applying the tracking information to the MRI data after it has all been acquired, whereas prospective correction involves adaptively adjusting the MRI pulse sequence in “real time” in response to motion, with low latency as the data are being acquired, attempting to ensure that the imaging field of view (FOV) remains aligned with the moving head. Although retrospective correction methods can often be more easily implemented because they do not require low latency or modifications to the MRI pulse sequence, prospective correction can rapidly and intrinsically provide clinicians with motion-corrected images, which is an appealing solution [2]. However, in scenarios where tracking is erroneous or slow and incorrect adjustments are made to the MRI pulse sequence, the resulting images may include new artifacts and uncorrected data are irrecoverable. The strengths and weaknesses of both retrospective and prospective correction continue to be researched to produce brain images with better motion artifact suppression.

Irrespective of whether motion correction is retrospective or prospective, accurate and robust motion tracking is of critical importance. Tracking can be accomplished using features of MRI data or various interleaved MRI measurements (e.g., navigator echoes [5]), which are beneficial in that they reduce the need for additional tracking equipment and intrinsically provide tracking data in the MRI coordinate system. However, these strategies have spatiotemporal resolutions which are limited by the physics of MR image formation and can suffer from issues involving static magnetic field and gradient field inhomogeneities and magnetic susceptibility, all of which reduce the quality of the tracking data. Alternatively, optical tracking (OT) has been investigated as a potentially useful alternative for many years. The benefits of OT include good MRI compatibility, very high accuracy (~5–100 μm), and high temporal resolution (~20–50 ms), without requiring MRI pulse sequence modifications to obtain tracking data [4]. Several groups have made use of OT systems, resulting in high-quality motion correction [6,7,8]. However, the drawbacks of OT continue to hamper clinical adoption, for example, the need for “line-of-sight” and adequate illumination, unequal performance across DOF, lengthy calibration times, and, in cases where OT involves the use of fiducial markers, insufficient rigid fixation of the marker causing relative motion between the marker and the brain [9].

Recently, deep learning methods for motion correction have grown in popularity and sophistication. These methods primarily focus on retrospective correction by image registration [10] or artifact removal, either via a convolutional neural network [11,12], or a general adversarial network [13,14,15], rather than by direct motion tracking. In parallel, there have been considerable advances in the use of deep learning for computer vision and estimation of the position and orientation (the pose) of rigid bodies in 6 DOF [16]. These computer vision approaches have the potential to overcome the drawbacks of traditional OT for motion correction in MRI but to date, this opportunity remains to be explored in detail. Of the deep-learning-based applications used to estimate head pose, most, if not all, focus on tracking large scale head motion, often to determine attention status and to track gaze [17]. For applicability in correcting head motion in MRI, tracking data must be 5–10 times more precise than the voxel size of the image, corresponding to a target accuracy of ~0.1–0.2 mm in OT for anatomical imaging applications [18]. Pose tracking tasks at a sub-millimetre scale have been typically focused on automation applications, such as manipulation of a robotic arm for fine placement of parts [19,20,21]. Thus, there remains a knowledge gap concerning the use of optical cameras to provide input to a robust deep neural network to track sub-millimetre head motion during MRI of the brain.

The focus of the present work is to address this knowledge gap by outlining initial proof-of-concept developments that both enable and demonstrate the potential utility of combining OT and deep learning technology for head motion correction in MRI. An MRI-compatible testing platform is designed and described, primarily constituting OT System 1. This system includes remote stereoscopic video cameras and a custom fiducial “marker” tracking tool for collecting ground truth pose information at high spatiotemporal resolution—suitable for supervised training of deep learning head pose tracking models. The testing platform is described in sufficient detail for others in the scientific community to replicate and augment as appropriate, and example tracking data are shown from a representative young healthy adult. Additionally, a hardware prototype is shown for a markerless, deep-learning-based OT system (OT System 2), consisting of two in-bore MRI video cameras with minimal setup requirements, followed by use of a convolutional neural network to estimate head pose based on the paired camera image data. Preliminary simulated tracking data are reported for OT System 2, demonstrating feasibility of the approach for clinical MRI, and motivating further acquisition of ground truth data using the testing platform.

## 2. Materials and Methods

**Figure 1:**
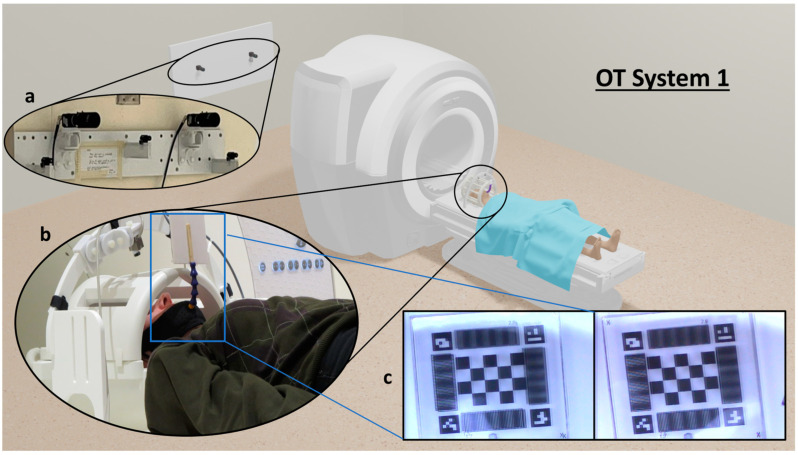
OT System 1. (**a**) Two cameras are mounted on the rear wall in the magnet room facing the magnet bore. (**b**) A participant lies in the head coil, who is fitted with a wearable device with a fiducial marker attached. (**c**) The marker is visible in each camera as displayed in the bottom right. OT = Optical Tracking. This figure includes 3D assets adapted from “Sci-Fi MRI” by Mihail Shegolev (https://skfb.ly/oESMO accessed on 6 May 2024) and “Human” by Aaron Kalvin (https://skfb.ly/6Z8LI accessed on 6 May 2024) licensed under CC BY 4.0.

The prototype testing platform for conducting ground truth motion tracking measurements (OT System 1) is illustrated in Figure 1. Two charge-coupled device (CCD) sensor video cameras (Watec WAT-204CX [22]) were mounted 125 cm vertically from the floor with a separation of 60 cm on the rear wall of a magnet room housing a research-dedicated 3 T MRI system (Biograph mMR, Siemens Healthineers, Erlangen, Germany). The cameras were oriented for stereoscopic tracking of an illuminated, custom fiducial marker in the centre of the magnet bore. To suppress effects of possible wall vibration (due to the adjacent MRI system equipment room), the camera mounts were made sufficiently sturdy, and the camera data were low-pass filtered in the frequency domain outside the range of measurable physiological motion. The distance between the cameras and the fiducial marker was approximately 3 m, depending on the head size of the patient or research participant. Each camera had an effective focal length of 200 mm (using a 100 mm focal length lens with a ×2 extender), a FOV of 76 × 58 mm, an image (frame) resolution of 720 × 480 pixels, and a temporal resolution of 30 frames per second (fps). All video data from the cameras were captured using open-source Open Broadcast Software (OBS version 28.1.2) [23]. OT System 1 was designed according to the criteria that the fiducial marker must be visible in the FOV of the cameras for the duration of imaging and must be highly accurate, with error < 1 mm in translations and <1° in rotations, across all 6 DOF.

### 2.1. OT System 1 Moiré-Based Fiducial Marker

#### 2.1.1. Marker Design Criteria and Features

A custom optical fiducial marker was designed for the testing platform for several reasons. First, the marker features needed to be scaled to appear distinctly in the FOV of the external cameras of OT System 1. Second, external position tracking systems often have variable performance across the 6 DOF, strongly dependent on the appearance of the marker. To satisfy the design criteria for OT System 1, careful design and fabrication work was required to ensure high-quality tracking data suitable for ground truth measurements.

A number of other research groups have achieved highly accurate OT during MRI [6,7,8]. Simple photogrammetric tracking of square-patterned fiducial markers (by comparing the measured distortion of the marker shape in the image and the known shape of the marker) is known to have poor measurement performance with “through-plane” DOF [24], where through-plane refers to any motion that does not occur in the plane that is parallel to the camera lens and passes through the centroid of the marker. Initial bench-testing with a single video camera confirmed that the inaccuracy in tracking through-plane DOF was considerable and unacceptable for ground truth tracking measurements in the present work. To overcome this deficiency, moiré patterns were added to improve through-plane rotational estimates, and two stereo video cameras were used to triangulate and improve the through-plane translational estimates.

**Figure 2:**
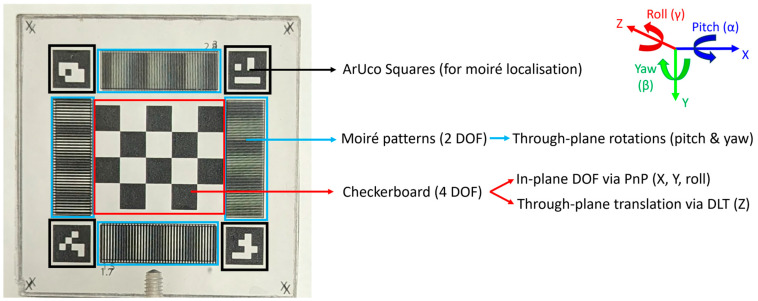
Design features of the moiré fiducial marker and the spatial coordinate system describing all 6 DOF. ArUco squares allow for localisation of the moiré patterns around the perimeter of the marker. The moiré patterns are used to measure the through-plane rotations. The central checkerboard is used to measure the in-plane DOF and through-plane translation via two algorithms (PnP and DLT). ArUco = Augmented reality University of Cordoba; DOF = Degrees of Freedom; PnP = Perspective-n-Point; DLT = Direct Linear Transform.

The design features of the fiducial marker are shown in Figure 2, as well as the spatial coordinate system describing all 6 DOF. A central checkerboard was used for tracking the in-plane DOF (translation in X and Y and rotation about Z (roll)) via calculating the solution to the perspective-n-point (PnP) problem [25]. The checkerboard was also used to track the through-plane depth (translation in Z) via the direct linear transform (DLT), which allows for triangulation of the points of the checkerboard in three dimensional (3D) space [26]. Four moiré stripe patterns were placed surrounding the checkerboard for through-plane rotation tracking of pitch (rotation about X) and yaw (rotation about Y). Augmented reality University of Cordoba (ArUco) squares [27,28] were placed in the corners of the marker to assist in localising the moiré regions prior to their analysis. ArUco squares are identified by contour detection algorithms and contain interior patterns, made of black and white squares in a 4 × 4 grid, for encoding an identification number to distinguish each square. This allows not only localisation of the corners of the marker but also the determination of each corner, and therefore the adjacent moiré pattern, uniquely. OpenCV [29] (version 4.6.0), a widely used open-source computer vision library, was used for the PnP and DLT computations (via the functions solvePnP and triangulatePoints, respectively), whereas NumPy [30] (version 1.21.5) and SciPy [31] (version 1.7.3) Python libraries were used for the moiré computations.

#### 2.1.2. Moiré Pattern Design and Marker Fabrication

Moiré patterns have been used successfully both in general computer vision and in MRI motion correction contexts [6,24,32,33]. Moiré patterns are an optical interference pattern that change appearance drastically when they undergo a small motion in relation to the observer. This property improves upon classical computer vision features (e.g., a checkerboard) which do not change in appearance appreciably under small motions.

The moiré patterns were created by layering two grating patterns with slightly different spatial frequencies [34]. These patterns were laser-printed on transparent sheets and carefully affixed to either side of a 10 mm thick substrate block of transparent polycarbonate, using liquid optically clear adhesive, cured with ultraviolet light. This layering created a “beat frequency” based on the interference of the two patterns. The result was an oscillating, thicker, striped pattern appearing through the printed region. When undergoing a small rotation, the moiré pattern showed a substantial phase change that was observed as a shift in the stripes.

The relationship between the printed grating frequency, separation between the grating layers, and the sensitivity of the moiré phase to motion was previously modelled [35]. Including an additional term for the refractive index of the substrate material, the equation is as follows:

(1) $$\theta =n\;sin\left(tan^{-1}\;\left(\frac{\varphi }{2\pi f_{1}d}\right)\right)\;,$$

where *θ* is the physical rotation in radians, *n* is the refractive index of the substrate (unitless), *φ* is the phase angle of the moiré pattern in radians, *f*_1_ is the spatial frequency of the print on the front surface (mm^−1^), and *d* is the separation of the two patterns due to the thickness of the substrate (mm).

The design of the moiré pattern was conducted with the goal of simple, cost-effective fabrication. The pattern was printed at the maximum resolution of a laser printer at 3 lines/mm (Xerox C60 Pro, Xerox, Norwalk, CT, USA). Although printing at a higher resolution would increase the sensitivity of the moiré pattern to rotations, only select printers can print on the transparent sheets used, thus limiting the resolution. The fabrication process could be improved for alternative applications where greater sensitivity may be desirable. However, for the present application the sensitivity was found to be sufficient (see Section 3). Two pairs of moiré patterns were printed: “fine” (3 lines/mm overlaid with 2.8 lines/mm), and “coarse” (1.7 lines/mm overlaid with 1.6 lines/mm). Each through-plane rotational DOF (pitch and yaw) used one fine and coarse moiré each to provide highly sensitive tracking over a broad dynamic range, in case of larger motions beyond the phase wrap rotation of the fine moiré.

The design constraint of moiré phase wrap in turn informed the selection of the thickness of the polycarbonate substrate, which was chosen such that the sensitivity of the moiré pattern was significantly greater than a 1° rotation per phase wrap. According to Equation (1), with a substrate thickness of 10 mm, the fine moiré pattern phase wrapped at 3.24° of rotation and the coarse moiré pattern phase wrapped at 6.31° of rotation. Therefore, to detect a 0.1° rotation, the fine moiré underwent an 8° phase shift and the coarse moiré a 4.5° phase shift.

**Figure 3:**
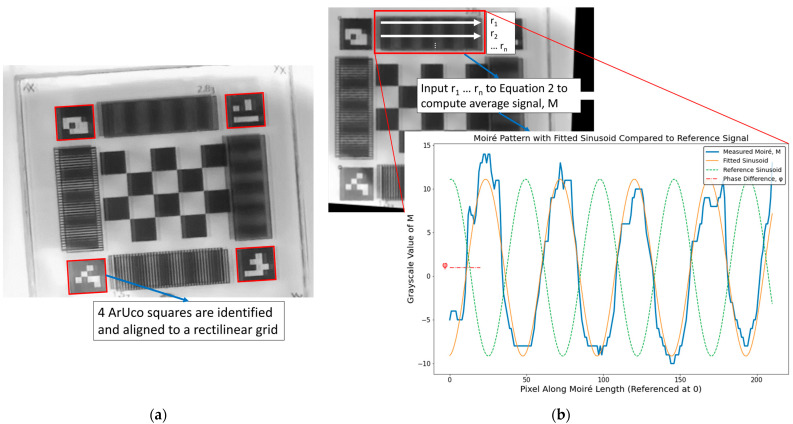
Moiré marker phase analysis pipeline. (**a**) The moiré marker is viewed in an arbitrary orientation and the ArUco squares are identified. (**b**) Based on the position of the ArUco squares, the marker is aligned to a rectilinear grid. The moiré waveform is averaged across rows or columns as appropriate (row averaging shown). This is repeated for each of the four moiré patterns. The average waveform is mean centred and fit to a sinusoid and compared to a reference to compute the phase. The phase value acts as input to Equation (1).

The moiré phase value *φ* was tracked for each of the paired patterns via the wall-mounted video cameras and input into Equation (1), along with the other marker parameters, to calculate the through-plane rotation of the marker in 2 DOF. Representative video frames and moiré data are shown in Figure 3. Prior to measuring the phase of the moiré, it was necessary to identify and isolate the moiré regions in the FOV of the frame. This was achieved using the ArUco squares in each corner of the marker. First, an image of the marker in an unknown orientation was captured (Figure 3a). Then, OpenCV was used to identify the location of the four ArUco squares using the detectMarkers function from the ArUco package in the OpenCV library. If one square was occluded then the moiré could not be assessed for this frame, and the frame was excluded from further analysis. Once the corners were identified, the fiducial marker was aligned to a rectilinear grid via a homography transform [26] based on the ArUco positions and their ideal positions on a flat faced marker model using the OpenCV functions findHomography and warpPerspective (Figure 3b).

Once the marker was aligned to a known grid, the regions of moiré had a defined pixel position. The four regions were extracted, and lines of grayscale pixel values were collected along the long edge of each region (Figure 3b) to create a set of sinusoidal patterns which were then averaged together, using the equation

(2) $$M=\frac{\sum_{i=1}^{n}r_{i}}{n}\;,\;$$

where *M* is the resultant average moiré signal for that region in grayscale values, *r_i_* are the lines of grayscale pixel values along the moiré length, and *n* is the number of grayscale lines acquired (unitless). This equation created a single sinusoidal representation of the moiré signal for each region.

The sinusoid was then fit to a standard sinusoidal curve of the equation

(3) p=Asin(ω∗s−φ)+b, 

where *p* is the fitted signal, *A* is the amplitude of the sinusoidal curve in grayscale values, *ω* is the spatial frequency of the moiré pattern in mm^−1^ (nominally equal to the beat frequency of the moiré pattern, but fit to the model to account for potential fabrication imperfections), *s* is the pixel position along the length of the moiré, *φ* is the phase difference between the sinusoidal curve and a reference curve in radians, and *b* is the offset in grayscale values.

This analysis pipeline, described in Figure 3, was conducted specifically to estimate the phase *φ* using the curve_fit function from the SciPy Optimize library. The estimated *φ* value was then input to Equation (1) to compute the physical rotation of the marker.

#### 2.1.3. Wearable Device

**Figure 4:**
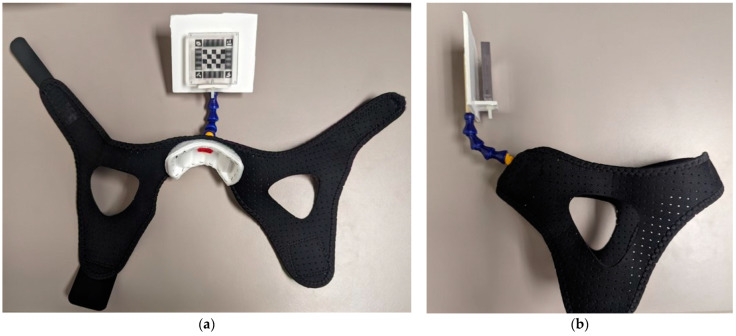
Wearable device with moiré marker. (**a**) Front view of unclasped wearable device. (**b**) Side view of clasped wearable device.

The wearable device was designed to extend out of the head coil opening above the neck and chin, out of view of the in-bore cameras, and to arc upwards holding the moiré marker facing down the centre of the bore towards the external ground truth cameras (Figure 1). The wearable device is shown in detail in Figure 4 with the marker attached. The wearable device featured two straps for support positioned on the top and the back of the head. Velcro™ on the straps was extended to improve stability and fit smaller heads. The hole created by the structure of the two straps created unobstructed space for the ears, which was necessary for comfort and for participants to use the headphones in the MRI system to communicate with the operator at the console. An adjustable, purple, plastic “gooseneck” was anchored into a cushioned plastic chin cup and used to position the marker in the narrow FOV of the external cameras despite variable head size across participants. Small, medium, and large versions of the wearable device were created with varying numbers of gooseneck links to accommodate different head sizes. Figure 4 shows the medium wearable device which was created with four plastic links. The small wearable device was created with two links, and the large device with five.

### 2.2. OT System 1 Camera Calibration

Although OT systems are desirable for their high accuracy, temporal resolution, and MRI-compatibility, lengthy setup and calibration concerns may reduce the practicality of using such systems in clinical applications. Although these lengthy procedures have been partially mitigated in some OT systems [36], deep learning approaches present another practical opportunity to minimise setup. However, to generate a training dataset for deep learning methods a ground truth system is necessary. OT systems are an excellent choice for determining ground truth, especially in a research and development context where lengthy setup proves less of a concern and where an efficient, highly repeatable calibration process can be devised for useful tracking data. This calibration process is described below, as developed by trial and error.

For OT System 1, calibration of the “intrinsic” camera parameters was required to remove lens distortion from the images; calibration of the “extrinsic” parameters was required to define the 6 DOF relationship between the pose of the two cameras, as essential for stereoscopy and triangulation. As changing the focus or zoom of the cameras would invalidate the calibration, the calibration needed to be undertaken in the magnet bore of the MRI system, where the two cameras were to be focused during operation [37]. The intrinsic and extrinsic parameters were calibrated efficiently in a single step by using the left and right external video cameras to capture sets of paired images of a calibration board (a black and white 10 × 15 checkerboard with a square size of 3 mm × 3 mm). The images were generated spanning the entire camera FOV to capture heightened distortion at the edges of the images, for multiple orientations of the calibration board.

To ensure accuracy, efficiency, and reproducibility of the calibration, the camera frame FOV was partitioned as an overlapping 3 × 3 grid. The calibration board was moved to each of the nine positions sequentially, pausing in each position to collect five calibration images for five angles (forward facing with no tilt, tilt left, tilt right, tilt up, and tilt down), respectively. After the ninth position, four more images were taken in the centre position with compound rotations (tilt to top left, top right, bottom left, and bottom right) and one more image was taken facing forward. The overall number of calibration images totalled 50. This procedure was determined via trial and error, with additional images being collected during preliminary investigations in instances where the calculated parameters were clearly incorrect (e.g., geometrically improbable extrinsic parameters) until it was determined that 50 images were sufficient. This extensive calibration procedure was necessary due to the high focal length of the lens (200 mm) leading to instability in the calibration calculation [38].

Moving the calibration board through these positions and orientations in the magnet bore would be irreproducible and prone to error if performed completely manually. A simple wooden stage was thus created to facilitate the procedure. The stage had a single 3.2 cm long horizontal slot cut in the centre and a vertical post with the calibration board attached on top. The post was attached to a long rod that extended outside of the bore for remote operation. When the post was inserted into the horizontal slot, the calibration board could be placed repeatably through the horizontal range of the FOV in three positions (left, right, and centre, by aligning with a centre marking along the slot). To cover the vertical range, the vertical post had three notches cut at 0.7 cm of separation (for 1.4 cm total length), which allowed the calibration board to be placed in three repeatable vertical positions in the FOV. The three notches were cut halfway around the circumference of the post with sufficient thickness to allow for rotations in both the horizontal and vertical directions. The calibration stage and post were moved to the centre of the bore at the focus depth of the camera where all 50 calibration images were taken. In combination, all nine positions were reached reproducibly with the apparatus. The calibration intrinsic and extrinsic parameters were then calculated using these image data and the OpenCV functions calibrateCamera and stereoCalibrate, respectively.

Examining the reprojection error is a common method of validating camera calibrations. The reprojection error, quantified as the difference in pixels between the real, measured calibration points and calibration points reprojected back into 3D space based upon the resultant calibration parameters [26], was measured for both the intrinsic and extrinsic parameters via the OpenCV calibration functions to assess calibration accuracy.

### 2.3. Validation of OT System 1

#### 2.3.1. Benchtop and in-Bore Pose Estimation Validation

Validation of the testing platform involved careful testing of how well the external cameras were able to track the custom fiducial marker in all 6 DOF, as well as the accuracy and reproducibility of the cross-calibration (thus providing tracking data in spatial coordinates of the MRI system). Initial validation testing was conducted by OT of the marker on the benchtop, and then in the magnet bore. The cross-calibration procedure was validated next, followed by an evaluation of the system capability to track head motion of a participant.

For the benchtop test, the marker was attached to positional and rotational stages (Newport Corp., Irvine, CA, USA, 423 and 481-A) capable of moving the marker in increments as small as 0.1 mm and 0.1°, respectively. The two video cameras were clamped to the benchtop, positioned 60 cm apart and rotated inward to view the stages clamped 3 m away on the other end of the benchtop. The room was well illuminated with fluorescent ceiling lighting. The cameras were calibrated as described previously although the calibration board could be easily manipulated by hand in this case, and therefore the wooden stage was not required.

The performance of the PnP method on all 6 DOF was initially tested to confirm its suitability for tracking in-plane DOF and inadequate performance for through-plane DOF in the present application. The marker was moved in increments of 0.1 mm and 0.5° for the translational and rotational DOF, respectively. The range measured was from 0–1.5 mm for translation and 0–25° for rotation. Next, the marker was tested to assess the performance of the DLT method on improving through-plane translational estimates and to assess the utility of the moiré stripes for improving the through-plane rotational estimates. The marker was moved in increments of 0.1 mm and 0.1° over a range of 0–2 mm and 0–2° for translations and rotations, respectively. At each position, the two ground truth video cameras were used to record a short 5 s (150 frame) video of the marker. The relative pose estimate of the marker for each pose increment was averaged across the 150 frames and the standard deviation and mean absolute error were calculated.

After benchtop validation, the marker was tested in the magnet bore to ensure that there was no significant degradation in tracking performance in the MRI environment due to change in illumination or other environmental factors. The cameras were mounted to medium density fibreboard (40 × 6 × ¾ inches) on two 3D-printed adjustable camera mounts. The board itself was mounted on the rear wall of the magnet room (Figure 1). Additional illuminators (generic light-emitting diode (LED) vehicle lamps modified for MRI-conditional use) were installed below the cameras, facing upwards into the magnet bore. The wooden calibration stage was used again to move the marker to known locations for tracking the measured change in position or orientation. Although the stage was not machined with millimetre accuracy, it was suitable for verifying tracking and enabled tracking of positions throughout the FOV. A second wooden stage, similar to the calibration stage but with a single slot, enabled the marker to be moved 1 mm forward and backward to assess depth (Z translation) accuracy. Video data of the marker moving through the full range of a single DOF at a time were collected across 3 trials. Peak-to-peak distance was measured for each trial and the mean absolute error from the known distance was calculated.

#### 2.3.2. Cross-Calibration between the Camera and MRI Coordinate System

**Figure 5:**
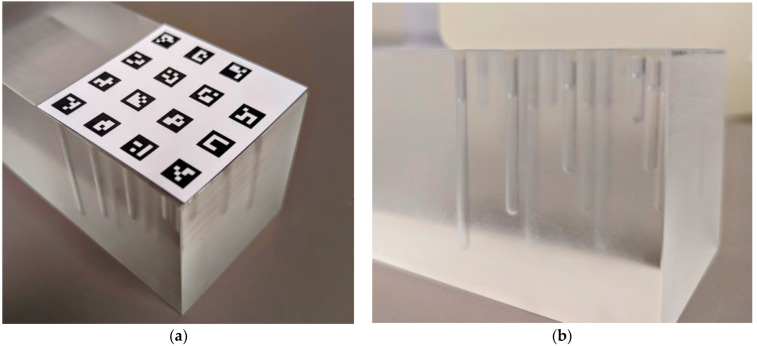
Cross-calibration phantom. (**a**) Camera-visible points using ArUco squares. (**b**) MRI-visible points as wells filled with copper-II sulfate solution. Camera-visible points are positioned directly above the MRI-visible points with a known difference in depth.

To be used for motion correction, the ground truth labels (position tracking measurements) for the training dataset need to be transformed from the spatial coordinate system of OT System 1 to that of the MRI system. This was achieved by simultaneous OT and MRI of a stationary “phantom” test object with a set of MRI-visible and camera-visible matched points, similar to previous workflows in other studies [7,9]. The transformation matrix between coordinate systems was then calculated via an effective singular value decomposition (SVD) method [39]. The phantom design is shown in Figure 5. The MRI-visible points were created by drilling 15 vertical wells into the body of the phantom and filling them with a 12.5 mM aqueous solution of copper-II sulfate (T1 = 88 ms, T2 = 66 ms). The camera-visible points were ArUco squares detected via the OpenCV library. The phantom underwent simultaneous MRI using a 3D T1-weighted magnetisation prepared rapid gradient echo (MPRAGE) sequence (repetition time/echo time/flip angle = 5.7/2.52 ms/20°, slice thickness = 0.75 mm, matrix = 256 × 256, FOV = 192 mm) as well as stereoscopic video recording to generate the set of matched points. The 15 ArUco squares were point-matched with the lowest depth of the 15 phantom wells. The precise distance between the point pairs was known, as outlined by the phantom design documentation, and confirmed by caliper measurements. Due to the possible changes in position of the phantom, not all 15 points were always concurrently viewed in frame. To compute the transformation matrix, only 3 points were required; thus, the loss of some of the 15 redundant points was acceptable.

The cross-calibration phantom was positioned within the head coil of the MRI system, and thus outside the normal FOV of the cameras for OT System 1. To alleviate this problem, an angled mirror (normally positioned at the top of the head coil for patients to view the MRI console area while lying in the magnet bore) was positioned facing the rear of the magnet bore, enabling the cameras to view the top of the phantom. The points as viewed through the mirror were rotated about the X axis by 90° in the coordinate system of OT System 1; therefore, the points were rotated −90° as an initial preprocessing step prior to calculating the transformation matrix. Several other known spatial relationships were also added in preprocessing: namely, the camera and MRI coordinate systems differed from each other by approximately 180° rotations about the X and Z axes, respectively; and there was a translation between the two coordinate systems of approximately 3000 mm in the Z direction. Ensuring that the cross-calibration accounted for the actual difference from these approximate values improved the stability of the transformation matrix calculation by limiting the rounding errors possible on large rotations or displacements.

The repeatability of the cross-calibration procedure was evaluated by computing the transformation matrix five times consecutively. For each of the trials, the phantom was moved to a slightly different position and orientation within the FOV of the cameras. The maximum error was calculated for each DOF to determine whether the error fell within the thresholds of 1 mm for translations and 1° for rotations—ideally with as low error as possible to enable motion correction in high resolution MRI.

#### 2.3.3. Initial Validation of Head Tracking Data

As a final validation step, the marker was rigidly attached to a representative young healthy adult participant via the wearable device to demonstrate motion tracking capability. The participant was instructed to hold their jaw in a comfortable, stationary position to not contaminate the tracking data with nonrigid jaw movement. A simple head motion was taught to the participant, with a focus on ensuring that the motion was not exaggerated and was as small as expected in MRI. For a duration of approximately 340 s, the participant made small, self-paced motions shaking their head “no” as they lay on the patient table with their head inside the head coil in the magnet bore, while OT System 1 recorded tracking data. The resulting data were assessed qualitatively on the ability to capture the recorded motion.

### 2.4. Prototype OT System 2 Development

#### 2.4.1. Synthetic Dataset Generation

After initial design and validation, the utility of the testing platform was demonstrated by proof-of-concept work related to prototype development of OT System 2. This system consists of two in-bore, MRI-compatible video cameras that track head motion based on deep learning, without the use of fiducial markers.

**Figure 6:**
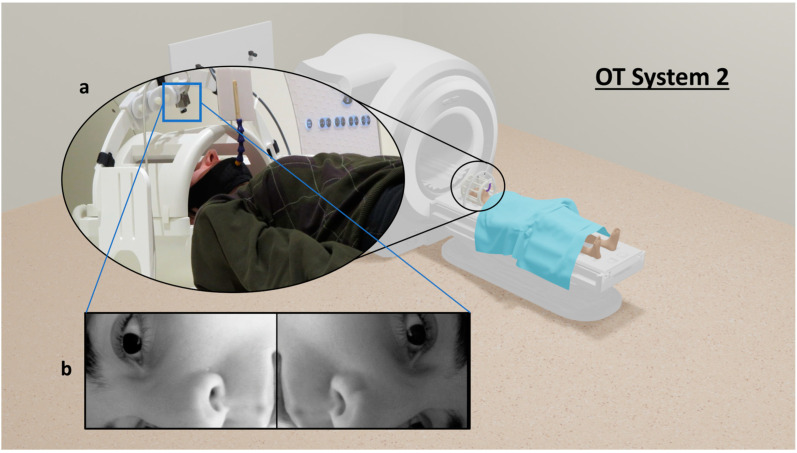
OT System 2. (**a**) Two in-bore, MRI-compatible cameras are positioned on an arch above the head coil. (**b**) The cameras point through the rungs of the head coil to view the left and right side of the face. OT = Optical Tracking.

The actual OT System 2 prototype is shown in Figure 6. Two MRI-safe complementary metal-oxide semiconductor (CMOS) video cameras (12M and 12M-I [40], MRC Systems GmbH, Heidelberg, Germany) available in the lab were attached to a 3D-printed arch mount positioned over the head coil to view the head through the coil rungs. Two video cameras were used rather than one, to provide richer information to the neural network by viewing the head at opposing angles. Each video camera had an effective focal length of 6 mm, a FOV of 151 × 112 mm, an image (frame) resolution of 720 × 480 pixels, and a temporal resolution of 30 fps. For the present proof-of-concept work, the arch was placed repeatably in the magnet bore by ensuring that the feet of the mount were equidistant to the end of the patient table. The calibration procedure for these cameras was conducted as outlined for OT System 1 but for each camera individually rather than as a pair, because these cameras were not operated stereoscopically. Each camera was calibrated to obtain estimates of intrinsic parameters only. This was carried out manually at the opening of the magnet bore, without using the wooden calibration stage.

The desired function of the neural network component of OT System 2 was to input four images and output 6 DOF values, with the first two input images consisting of the two in-bore camera views at an initial time point and the second two images consisting of the same camera views at a subsequent time point. The output 6 DOF values were chosen to represent the change in pose between the two timepoints because for the vast majority of MRI applications, successful motion correction relies on tracking the incremental change in pose, not the absolute pose.

A synthetic dataset of head images was generated for the purposes of pre-training a deep neural network and as proof of concept. Pre-training deep neural networks is a powerful method that enables high performance by training the model to identify desired low-level features on a similar dataset prior to fine-tuning on more complex, real-world data. Datasets for pre-training, a type of transfer learning, can be found via open-source methods, such as the ImageNet dataset; however, utilising a dataset of synthetic images mimicking the expected data distribution can provide a better estimate of performance and teach more relevant features to deep neural networks.

**Figure 7:**
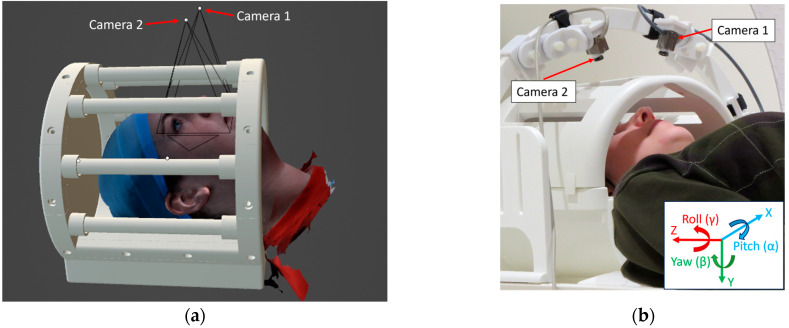
Comparison between the simulated environment and the real-world environment. (**a**) The simulated environment in Blender 3D modelling software. Simulated camera sources and their FOV are displayed. (**b**) The real-world environment. The reference coordinate system for both the simulated and real environment is shown in the bottom right.

**Figure 8:**
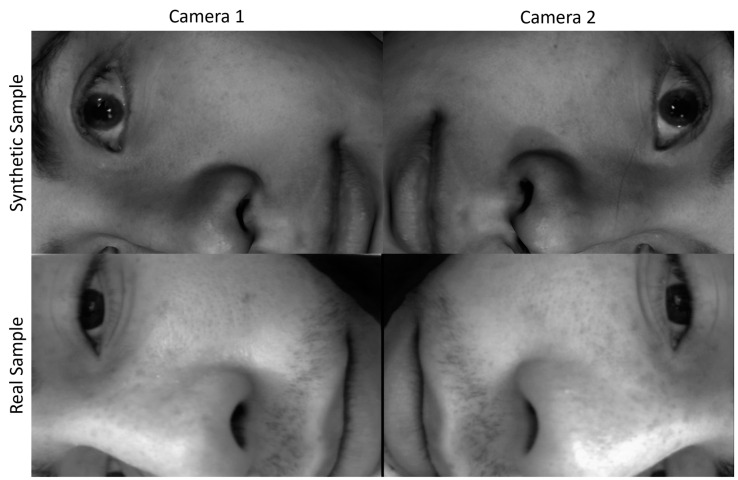
Comparison between a synthetic image sample (**top**) and a real-world sample (**bottom**) viewed through both in-bore cameras.

Using open-source Blender 3D modelling software (version 3.5) [41], a simulated environment was created featuring a head coil, two cameras, light sources, and highly realistic model heads from the Headspace dataset [42]. A comparison between the real-world and simulated environment is shown in Figure 7. Settings for the simulated cameras were matched as closely as possible to the in-bore video cameras with a focal length of 6 mm, sensor size of 4.1 mm, depth of field of 50 mm, and a F-stop value of 2.4. The head model was placed within the head coil in a supine position and at a “realistic” depth, such that the shoulders of the model were touching the bottom of the coil in most cases. Synthetically generated images and real-world sample data are shown and compared in Figure 8.

Head motion was simulated by generating six noisy sinusoids for each of the DOF. The sinusoids had an amplitude of 1.5 mm and 1.5°, a randomised starting point (phase shift), and a randomised frequency ranging from [1.7, 2.3] Hz to mimic the expected amplitude of common head motion in MRI [43]. Note that the upper frequency bound was chosen to be slightly greater than that of head motion expected in MRI so that the generated dataset covered a larger variety of positions for training. The sinusoids were of 67 s duration with a sampling rate of 30 Hz, corresponding to the 30-fps rate of the actual video cameras, for a total of 2000 samples per sinusoid. Each sample represented two frames (one from each camera) at a single head pose. All 6 DOF of the head model were updated simultaneously during the motion simulation, with each DOF altered slightly in each subsequent frame.

#### 2.4.2. Pre-Training and Proof of Concept

**Figure 9:**
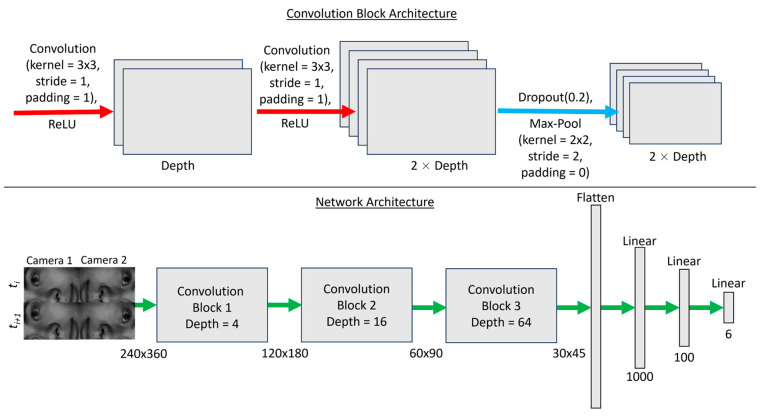
Outline of the simple convolutional neural network for prototype OT System 2. The network architecture is shown on the bottom and the contents of the convolutional blocks are shown on top. Each convolutional block had an increasing depth (4, 16, 64) and contained two convolutional layers (red arrows) (kernel = 3 × 3, stride = 1, padding = 1) with ReLU activation, a 20 % dropout layer, and a max-pool layer (blue arrow) (kernel = 2 × 2, stride = 2, padding = 0). ReLU = Rectified Linear Unit activation.

In this initial work, only one of the 50 head models was used to illustrate proof of concept for OT System 2. A basic regression convolutional neural network was created which output the estimated 6 DOF. The convolutional network was created in PyTorch [44] with layers and dimensions as outlined in Figure 9. A learning rate of 1 × 10^−4^ was used with the Adam optimiser, and a weight decay of 1 × 10^−5^ was included as a regularisation term. Kaiming weight initialisation was used and mean squared error (L2) was used as the loss function. A dropout of 0.2 was used once per convolutional block for regularisation. The model was trained for 100 epochs, or training loops. The dataset was split into training, validation, and testing sets with 1400 poses in training, 400 in validation, and 200 in testing. Distributions of all three datasets were compared via their mean, median, standard deviation, minimum value, and maximum value for each of the 6 DOF for consistency.

Isolation of the testing set was ensured throughout data pre-processing steps, whereas the training and validation datasets were pre-processed together. The pre-processing steps were as follows for the input images: scaling grayscale pixel values of [0, 255] to [0, 1], resizing the images to 180 × 120, and converting to the Tensor data type. No data augmentation was performed, as many image data augmentation steps (rotating, translating, shearing, etc.) common in computer vision would negatively impact a model learning fine rotations and translations in an image. Data pre-processing on the labels included a standardisation procedure where each DOF was rescaled to a range [0, 1] based on the minimum and maximum value found in the training and validation sets. This was accomplished using the MinMaxScaler function in the scikit-learn library [45] (version 1.0.2). The scaler mapping was saved and at inference time the model output was unscaled. Testing inputs were scaled based on the mapping obtained from the training and validation sets. Prior to training, the training and validation data were shuffled, whereas during testing the data were not shuffled. After the trained model was evaluated on the 200 samples set aside for testing purposes, it was also tested on 200 samples of a head model unseen during training, to assess generalisability.

## 3. Results

### 3.1. OT System 1 Benchtop Validation

Table 1 shows the results of position tracking at the benchtop as given by the mean measured position and standard deviation across 150 sampled frames. The method of measurement is specified in brackets with each DOF, where PnP is the solution to the perspective n-point problem, DLT is the direct linear transform, and moiré refers to the custom pipeline described in the methods above. It is evident that the PnP approach, obtained with a single camera, was sufficiently accurate for in-plane translation and rotation of the marker (i.e., X, Y and Roll, respectively). As indicated in the table, the reported mean measured position for the Z translation using PnP is misleading. Although the mean appears to closely match the real position increment, the measured value across the 150 samples varied greatly due to the instability of the PnP solution. The direction of the measured position oscillated between positive and negative values with variable amplitudes, leading to an overall average close to zero and coincidentally near the expected value. The median is reported below the table, which in combination with the high standard deviation more clearly illustrates the inaccuracy of this method in estimating the Z translation.

The inaccuracy in through-plane Z translation as measured by PnP calculations was avoided using the DLT method with stereo cameras, and the inaccuracy in through-plane rotations (Pitch and Yaw) as measured by PnP calculations was avoided using the moiré measurements.

### 3.2. OT System 1 Camera Calibration Results

The accuracy of the intrinsic camera parameters for undistorting images directly affects the accuracy of the extrinsic camera parameters. Therefore, Table 2 lists the estimates for the extrinsic calibration parameters of the cameras in OT System 1, indicating the position and orientation differences between the two when mounted in the magnet room. Note that the camera on the lefthand side (when facing towards the magnet bore) was at the origin of the spatial coordinate system, and that the listed parameters thus indicate the transformation required to align the right-side camera to the origin. The extrinsic parameters produced were as expected. The nominal distance between the two cameras was measured as 60 cm. The cameras, positioned on either side of the magnet bore, were rotated inwards creating an angular difference of approximately 10°. Given that the extrinsic parameters were well estimated, the intrinsic parameters were inherently validated as well.

The reprojection error for intrinsic camera calibration, averaged based on the difference in pixel positions of the corners of the calibration checkerboard, was 0.206 and 0.196 pixels for the left and right cameras, respectively. Reprojection error for the extrinsic camera parameters was 2.04 pixels. This higher reprojection error for the extrinsic camera parameters contributed to the higher Z extrinsic parameter estimated at 9.77 cm; the expected value is approximately 5 cm, caused by the inward rotational difference of the cameras. However, it was evident in the in-bore validation results (see below) that this had little impact on performance at ~3 m distance from the marker.

### 3.3. Validation of OT System 1

#### 3.3.1. In-Bore Pose Estimation Validation Results

The marker was moved in the bore to known positions as defined by the limits of the slot size and post height of the wooden stages. The marker was moved back and forth between the extremes of each position, and in each case held stationary for approximately 5 s while position tracking measurements were undertaken. Table 3 lists the results for these measurements indicating the actual change for each DOF, and the mean change and associated standard deviation based on the tracking measurements at the extrema. In each case the standard deviation was an acceptably small fraction of the mean, and the mean change closely matched the actual change in position.

#### 3.3.2. Cross-Calibration Validation Results

Table 4 lists the results of the cross-calibration procedure. Over the five trials conducted, the maximum error for each 6 DOF was found to be 0.55 mm, 0.43 mm, 0.83 mm, 0.69°, 0.90°, and 0.70° for X, Y, Z, pitch, yaw, and roll, respectively. All of the maximum errors were under 1 mm and 1°, indicating suitability for motion correction. Note that the transformation parameters reported are to be applied to camera tracking data after the data are rotated about X and Z by 180° and translated in Z by 3000 mm. The remaining transformations align the coordinate frames after these large discrepancies have been accounted for. The translational transformations were of the expected size, as the origin of the camera coordinate system was not in line with the magnet bore axis and thus the MRI coordinate system origin.

#### 3.3.3. Example Head Tracking Data

**Figure 10:**
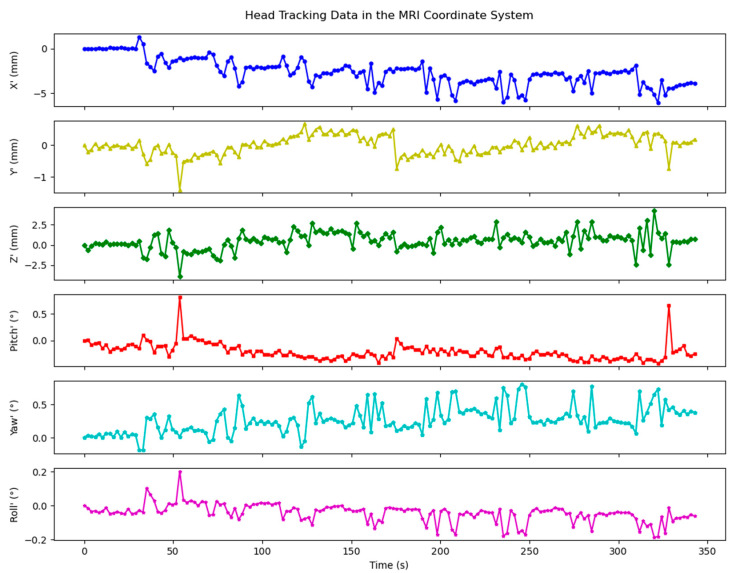
Initial position tracking data of a participant in MRI system coordinates. DOF in the MRI coordinate system are represented as X’, Y’, Z’, Pitch’, Yaw’, and Roll’.

Figure 10 shows initial position tracking data in MRI system coordinates of a representative young healthy adult participant undergoing a self-paced shaking “no” head motion (yaw). Intentional motion began at 28 s; cues for motion to start and stop occurred every 20 s thereafter, totalling eight periods of motion across ~340 s. Six different plots are shown, each with the vertical axis adjusted to reveal the maximum range of pose effects for a specific DOF. Complex head motion was recorded, capturing the effects of this head motion across all DOF. The patterns did not show a pronounced “noise floor”, as might be expected if the cameras were recording motions at the detection limit; instead, adequate dynamic range was displayed to track real motions well below 1 mm and 1° in translations and rotations, respectively.

### 3.4. Prototype OT System 2 Pre-Training Results

The initial pre-training results for OT System 2 are listed in Table 5 based on simulation data for a single head model subjected to known, random head motions with physiologically appropriate characteristics. Results from training, validation, and testing sets are provided, including testing on an alternative head model that was unseen by the network during training. Results are listed as the square root of the average mean squared error ($\sqrt{MSE}$) to represent uncertainty in position tracking in the measurement units for each DOF. Note that although increased $\sqrt{MSE}$ values were obtained when testing on an unseen head model, in comparison to those for the head model used in training, all values were well below 1 mm and 1° for translational and rotational DOF, respectively. This increase is expected because the unseen head model has differing facial characteristics, skin tone, etc. than the known head model. Additionally, the change in performance for some DOF (e.g., Y and pitch measurements performed well on the known head but were the worst performing DOF for the unseen head) was likely due to changes in the distribution of motion characteristics for the unseen head model in comparison to the known head.

The error of OT System 2 was greater than OT System 1, as expected, when comparing the results between Table 1 and Table 5. In particular, the $\sqrt{MSE}$ of OT System 1 averaged separately across the three translational and three rotational DOF was 0.008 mm and 0.007°, respectively. This was notably lower than the $\sqrt{MSE}$ of OT System 2, which using the analogous averaging was 0.14 mm and 0.11° for the known head and 0.38 mm and 0.33° for the unseen head, respectively.

## 4. Discussion

The present work has taken important initial steps to address the current knowledge gap in OT and deep learning capabilities relating to head tracking for MRI applications, and to demonstrate the potential utility of this technology. Although current OT systems boast many advantages, they lack the robustness that deep-learning-based approaches can potentially offer, such as tolerance to varying facial expressions, varying illumination, and more uniform tracking performance across all DOF. Therefore, a testing platform comprising OT System 1 was developed and validated in its ability to measure ground truth motion parameters in the magnet bore. In addition, synthetic pre-training was conducted to demonstrate OT System 2 in proof of concept, via deep learning. These simulations were performed with closely matching operational parameters to the experimental, prototype hardware. These early simulations demonstrated promising results, supporting the feasibility and importance of conducting future work using the testing platform for full development of OT System 2.

The measurement of ground truth motion parameters is necessary to create an appropriate dataset for use in supervised training of OT System 2. Building off the previous experience of others [6,32,33], a moiré-based optical fiducial marker was designed in the present work to both utilise the strengths of marker-based OT and to improve the accuracy of through-plane DOF estimation. These improvements will help to ensure that OT System 2 will perform more equally in all DOF, as the system can only perform as well as the training dataset provided.

To emphasise the need for these improvements, the marker was tested on the benchtop using only the PnP method via the corner points on the central checkerboard. The results showed that there was instability in the PnP estimates of both through-plane rotations and translation. The estimated pose fluctuated between several values, indicating that the ambiguity of the image could lead to various solutions of the PnP problem. This performance limitation is well known in monocular tracking technologies, but it was particularly apparent in this instance due to the high focal length of the tracking cameras, which was necessary to achieve the zoom required to measure small changes in pose at a remote distance of ~3 m, outside the magnet bore. Focal lengths that result in a FOV narrower than 10° are known to experience difficulty in estimating orientation [38]; for context, the FOV of the OT System 1 cameras was approximately 1.5°. Strategies to enable more accurate calibrations of high focal length lenses in close range photogrammetry have been explored by others [38], but are complex enough that implementing such strategies was beyond the scope of the present work.

To improve the accuracy of through-plane DOF estimates, stereovision and moiré patterns were used. First, by using stereovision the through-plane translation along the Z axis was successfully triangulated with an accuracy as small as 0.1 mm, meeting the requirements for motion correction in MRI. Second, by using moiré patterns, changes in the through-plane rotations as small as 0.1° were detectable. Both results demonstrate superior tracking over the monocular PnP method and established the suitability of the marker for ground truth motion tracking.

Calibration of OT System 1 initially proved to be challenging because the calibration board had to be positioned in the centre of the bore within focus of the tracking cameras. Doing this consistently and repeatably by hand was laborious due to the small FOV of the cameras, making unoccluded positioning of the board both time consuming and impractical. By creating the calibration table and post, the resulting workflow was easier and much more repeatable. A potential future refinement of OT System 1 would be to create a more precisely manufactured table and post.

The calculated extrinsic camera parameters were found to match the real-world relative position of the two cameras, and the intrinsic camera calibration parameters were inherently validated as well. Accuracy was also assessed by inspecting the reprojection errors resulting from the calibrations. The intrinsic reprojection error was <1 pixel (excellent) but the extrinsic reprojection error just exceeded 2 pixels (acceptable although not ideal). The latter value is attributable to the extremely long focal length of the camera causing uncertainty during the calibration process. The tracking test results are dependent on the quality of the calibration and demonstrated high accuracy both in and out of the magnet bore, and subsequently the accuracy of the calibration did not hinder performance of the marker.

To confirm the performance of the marker in the magnet bore, the calibration stage and an alternative stage with a slot in the Z direction were used to move the marker to known locations. These stages were not manufactured with sub-millimetre accuracy, but they could be used to move the marker to known positions and to confirm that environmental changes in image quality would not affect pose estimation. Overall, changes in pose were found to be recorded with high accuracy.

Cross-calibration between the camera and MRI coordinate frames is a critical step in using external OT systems in MRI for motion correction, irrespective of whether retrospective or prospective correction is implemented. A custom phantom with camera- and MRI-visible paired points was used to compute the transformation matrix between coordinate systems [7,46,47]. Cross-calibration using the phantom was validated by performing five trials with the phantom moved to a different position in each case. The cross-calibration was highly repeatable, with a maximum error of 0.83 mm in the Z translation across the five trials. Despite this error taking up most of a millimetre, it has been noted in other studies that while the accuracy of the cross-calibration is paramount, the error can be on the order of a few millimetres across all DOF and still produce good motion correction results [9], supporting sufficient accuracy of the cross-calibration in the present work. Furthermore, compelling pilot data were subsequently obtained for a representative young healthy adult performing small, complex head motions while lying in the MRI system.

OT System 1 was meant to support the development of new position tracking technology, such as OT System 2. OT System 1 is inherently a prototype not intended for clinical use and could be improved by making several refinements in the future. It is likely that accuracy would increase by use of higher fidelity video cameras and more rigid, precisely fabricated stages for calibration in the magnet bore. The moiré patterns were made very economically and would benefit from future improvements in fabrication using precision masking and placement on the plastic substrate. In addition, future work could be conducted to optimise experimental workflow related to placement of the LED illuminator and placement of the marker to reduce marker glare. The illuminator was found to be essential, as lighting within the magnet bore was insufficient otherwise, but this came at the expense of unwanted reflection of light into the cameras (glare). Glare would sometimes obscure features on the marker with a negative impact on tracking. An anti-glare coating might be possible on the marker to remove glare as a confounding factor. Furthermore, the wearable device, although made to attach the marker to the head as rigidly as possible while simultaneously positioning the marker within sight of the wall-mounted cameras, could experience nonrigid motion of the jaw joint and thereby introduce errors into the tracking data. For technical development and the collection of the ground truth dataset, participants were instructed to keep their jaw stationary to avoid this. However, future modifications of the wearable device may alleviate this practice and would allow a wider range of participants to be included in the ground truth dataset. For example, if a head coil without a closed end was adopted, a hat design could be used instead of a chin strap design, with the marker positioned on the top of the head facing towards the cameras. This would move the marker out of view of the participant and would be less distracting, would not be prone to joint movement, and would likely be more comfortable to wear. The current implementation is functional for the present work, but researchers who may seek to replicate the present findings may find better utility in an improved design.

Turning to prototype OT System 2, the tracker was pre-trained on a synthetic head model undergoing various shifts in pose in a simulated MRI environment. The head was programmed to move in a manner mimicking the amplitude and frequency of physiological motion. The results of the initial proof-of-concept training to estimate relative change in pose across the 6 DOF between subsequent frames demonstrated high accuracy at the sub-millimetre and sub-degree level. The model was subsequently tested on samples from an unseen head to determine the performance and generalisability of the model. Performance for the X and roll DOF were the highest, indicating the capability of deep learning methods to estimate both in- and through-plane DOF well. Performance in the Y DOF was the poorest during testing, despite excellent results during training and validation. A possible explanation for this could be a quirk in the data split between training, validation, and testing. Although care was taken in ensuring equal distribution in each DOF across the three datasets, the distribution may not have been equal across combinations of DOF, leading to poorer performance in some DOF than others. This would be mitigated by including more samples in the dataset of a variety of different head models and head motion combinations.

The accuracy of the prototype model is likely attributable to several factors. Firstly, the FOV of the cameras is such that approximately 4.5 pixels span a millimetre, allowing for sub-millimetre tracking when detecting changes in single pixels. Standard photogrammetric techniques, such as those used to estimate PnP, often track at the sub-pixel level, which may be replicable with the deep-learning-based method and may further improve accuracy. Next, the face spans the entire FOV of the cameras, maximizing the size of facial features which are often the focus of deep learning models for facial recognition and head pose estimation [16]. Also, using two camera views as input to the network is beneficial as it provides richer data of the full view of the face, rather than a subregion. These factors are likely the driving force behind the performance observed for OT System 2 and should continue to be leveraged in future iterations—for example, by training on many faces with a variety of facial features and shapes that can be tracked.

When comparing OT Systems 1 and 2, the marker-based system boasts lower error than the markerless system. However, to achieve the superior performance shown by OT System 1, moiré and stereovision enhancements and lengthy calibrations were needed. Conversely, the operation of OT System 2 was more straightforward, did not require such enhancements or calibrations, and still achieved the required sub-millimetre and sub-degree accuracy. OT System 2 has yet to be tested on real-world motion, but it is anticipated that the error should increase the further the input data deviates from the simulation. It is certainly possible that in such a scenario it would no longer be possible for OT System 2 to meet the accuracy required for MRI motion correction. However, additional pre-training and ground truth training with more data points could lower the error of OT System 2 and bring it closer in line with OT System 1, making OT System 2 a true improvement to the marker-based system.

The synthetic head data were useful for pre-training OT System 2, and when fully deployed in the future will reduce the need for very large quantities of data to be collected in vivo for training purposes. However, such simulation models can also be unrealistic, as they do not undergo skin deformations (e.g., wrinkling, making facial expressions, or blinking). Deep learning models are known to be robust to these types of changes when they are included in the data, but this cannot be fully leveraged with synthetic data—it must be developed during final training with collection of the real, ground truth data. Another unrealistic condition is the lighting and shading of the synthetic models, which were implemented under ideal conditions to ensure that the head models were of high quality. Data augmentation steps can be taken in the future to change the brightness and contrast of images to create more variability, although the shadows on the models would remain immutable. Changes in shadows across the face will need to be captured by the real dataset, as these may be important features to track for inferring changes in head pose.

Another noteworthy issue is that the labels of the training datasets were provided with respect to the MRI coordinate system, as is necessary for motion correction and subsequent reduction in MRI motion artifacts. OT System 2 intrinsically learns the MRI coordinate system during training, without the need for cross-calibration measurements and associated error from incorrectly estimating the coordinate transformation matrix. However, if the in-bore cameras of OT System 2 were to move or change in calibration, the trained intrinsic model of the coordinate system could become inaccurate. To combat this in the present work, the arch mount for the in-bore cameras was positioned repeatably with respect to the end of the patient table. The measurements were not accurate to less than a millimetre but were sufficient given that the cross-calibration itself can withstand some degree of inaccuracy while enabling good motion correction. During data collection and training, if it is found that this method of repeatably placing the arch is insufficient, then a clamping mechanism can easily be developed that ensures consistency. OT System 2, in its current proof-of-concept form, also will not be MRI system-agnostic because it will be constrained to learning the spatial coordinates of the MRI system from which the training dataset is generated. Addressing this problem is beyond the scope of the present work and is reasonable to leave until the capabilities of OT System 2 have been better established, based on the ground truth data obtained from a single MRI system.

The prototype OT System 2 shows that the chosen design will likely have a robust, simple workflow during clinical MRI when compared with traditional marker-based methods. Time-intensive set up and calibration steps required for marker-based tracking, as demonstrated through the methodology of OT System 1, are not required for OT System 2, which only requires a simple, one-time calibration for undistorting images and simple placement of cameras in the bore. Additionally, the use of fiducial markers is often a barrier to clinical adoption due to the difficulty of affixing the marker rigidly to the head, especially in patient and pediatric populations. This problem is circumvented in the design of OT System 2. The markerless, deep-learning-based system is also advantageous compared to OT System 1 because it does not require “line-of-sight” to a marker, is more robust to changes in illumination, and likely has more equal performance in all DOF. Furthermore, with its robust design, OT System 2 could provide clinical utility beyond that of enabling high-quality motion correction. For example, OT system 2 could also be used to characterise the types, extent, and prevalence of motion in different patient populations, towards optimising how MRI motion correction is implemented and deployed.

However, some technical challenges associated with OT System 2 need consideration with future clinical adoption in mind. MR-compatible technology is costly, and the cameras used in this work cost approximately CAD 10,000 per camera. This is not unreasonable with respect to the typical cost of third-party MRI devices but may remain a sales and marketing challenge in some circumstances. Although the prototype is simplified in comparison to the marker-tracking camera setup, the in-bore cameras are not positioned optimally. They are mounted around the head coil with cables running out the front of the bore and around the outside of the magnet to the back wall, where the cables plug into signal filtering boxes for display and recording on the computer. The cables themselves must be monitored to avoid catching on the moving patient table, to avoid creating conductive loops which can create problematic currents during imaging and require some additional padding to separate the patient from the cables which could touch their body and potentially cause tissue heating. These are all tasks that would have to become an additional part of MRI technologist workflow, and need to be eliminated by simplifying the setup once the prototype is validated. Furthermore, as with all deep learning models, substantial testing in the clinic would need to be carried out to prove effectiveness and ease of use before integrating OT System 2 into clinical workflow. Deep learning methods are also known to suffer from biased performance based on items present in the training dataset. Although steps can be taken to ensure well-distributed and diverse data are used for training, extensive investigation should be carried out in the clinic to test for and eliminate biased performance of the model. The challenges discussed here are not exhaustive and it is anticipated that additional hurdles will be discovered during development and technical and clinical testing. Despite this, the outlook for OT System 2 remains very promising.

Future work in pre-training OT System 2 includes using more diverse synthetic heads (encompassing a range of skin tones, facial features, sexes, and ages) and training on a variety of network architectures. Expansion of the synthetic dataset to encompass diverse head models will help to mitigate model bias prior to testing on actual human data and will reduce the sample size required for such tests. The model trained here used a restricted dataset of one head model to demonstrate proof of concept and motivate the further development of this system with a larger dataset. The current model performed reasonably on an unseen head model during testing, but performance using this model is expected to degrade when exposed to a larger variety of head models and motion patterns. Development and training on a larger synthetic dataset will improve model robustness and generalisability in these scenarios.

Alternative neural network architectures have the potential to leverage different aspects of the dataset that could potentially lead to more accurate tracking. For example, because the goal of the model is to determine the change in pose between sets of video frames, a twin neural network could be used [20]. Such networks are often used in applications assessing similarity between images, which is highly pertinent to this application. Recurrent neural networks and other related networks may be able to leverage the time series sequence itself to better estimate head pose. Multiple networks will be trained and tested on the synthetic dataset and the highest performing model will be chosen for training on the real-world dataset.

Finalisation of OT System 1 and its set up procedure will enable real training data to be collected while OT System 2 undergoes further exploration and pre-training, such that OT System 2 is prepared for final training coincident with the completion of data collection. Training on the real-world dataset will incorporate strengths of the deep learning method that have yet to be leveraged, such as robustness to facial expressions and skin deformations, which are often limitations in OT systems. Also, despite the synthetic samples using realistic lighting and camera parameters, training on real-world samples will ensure that inaccuracies in these parameters during simulation will not affect model performance. As with all deep learning methods, training and testing on data that would be characteristically seen during deployment and clinical use is a critical step prior to implementing motion tracking and achieving motion correction.

## 5. Conclusions

The present proof-of-concept work describes development of a testing platform constituting an accurate marker-based tracking system, OT System 1, essential for the development of new tracking technologies to correct motion artifacts in MRI, as exemplified by markerless OT System 2, which incorporates deep learning. Markerless tracking methods continue to grow in popularity and sophistication, such as the success of the Tracoline system [36,48], and expanding upon this work with deep learning technology is a novel contribution towards sub-millimetre and sub-degree head pose estimation for robust and practical implementation, broadening the “toolbox” of available MRI motion correction methods. OT System 1 will enable the collection of ground truth head pose labels from test subjects (healthy participants, as well as patients) with high accuracy using a custom moiré marker, for the purpose of training the deep learning model which operates on the markerless, paired video images of OT System 2 as inputs. Progress on pre-training a deep neural network model using a synthetic dataset for OT System 2 indicates excellent feasibility of the approach.

OT System 2 will improve upon pre-existing marker-based solutions by providing more robust tracking not prone to issues of marker occlusion, variable illumination, and unequal performance across DOF. The markerless system will simplify hardware setup, be more comfortable for patients, and eliminate lengthy calibration routines, allowing for easier adoption into clinical settings. Work remains on expanding pre-training, collecting the ground truth dataset, and training and testing the final model, ultimately to enable highly accurate retro- and prospective motion correction for MRI of the brain.

## Figures and Tables

**Table 1:** Benchtop fiducial marker test results. PnP = Perspective-n-Point; DLT = Direct Linear Transform; DOF = Degrees of Freedom.

Translation DOF	Real Position Increment (mm)	Mean Measured Position Increment (mm)	Standard Deviation (mm)	Rotation DOF	Real Orientation Increment (Degrees)	Mean Measured Orientation Increment (Degrees)	Standard Deviation (Degrees)
X (PnP)	0.1	0.09	0.01	Pitch (X) (PnP)	0.5	0.01	1.83
Y (PnP)	0.1	0.10	0.04	Yaw (Y) (PnP)	0.5	0.05	2.21
Z (PnP)	0.1	0.13 *	1.39	Pitch (X) (Moiré)	0.1	0.095	0.04
Z (DLT)	0.1	0.11	0.02	Yaw (Y) (Moiré)	0.1	0.1	0.02
				Roll (Z) (PnP)	0.5	0.49	0.13

* Reported mean was not representative of typical performance, as is indicated by the high standard deviation. For comparison, the median PnP Z estimate was 0.49 mm.

**Table 2:** Extrinsic camera calibration results.

Unit	Degree of Freedom	Extrinsic Parameters
mm	X	−609
	Y	−3.11
	Z	−97.7
degrees	Pitch (X)	−0.27
	Yaw (Y)	9.59
	Roll (Z)	−1.07

**Table 3:** In-bore measured change in position compared to real change in position.

Unit	Degree of Freedom	Actual Change	Mean Measured Change	Standard Deviation
mm	X	23	21.8	0.6
	Y	17	16.9	0.2
	Z	1	1.1	0.5
degrees	Pitch (X)	5	5.3	0.2
	Yaw (Y)	11.5	11.3	0.4
	Roll (Z)	20	19.8	0.3

**Table 4:** Cross-calibration results across five trials.

	Trial Number	Pitch	Yaw	Roll
Rotation (degrees)	Trial 1	−5.09	0.09	4.54
	Trial 2	−5.02	0.41	4.67
	Trial 3	−5.36	−0.018	4.127
	Trial 4	−5.57	0.144	4.78
	Trial 5	−5.19	0.25	4.96
	Mean	−5.25	0.18	4.62
	Standard Deviation	0.20	0.15	0.28
	**Trial Number**	**X**	**Y**	**Z**
Translation (mm)	Trial 1	−6.65	−18.2	−67.1
	Trial 2	−6.19	−18.1	−67.3
	Trial 3	−6.65	−18.9	−67.7
	Trial 4	−5.96	−18.6	−67.7
	Trial 5	−6.11	−19.0	−67.8
	Mean	−6.31	−18.56	−67.52
	Standard Deviation	0.29	0.36	0.27

**Table 5:** Initial pre-training results for OT System 2.

$\mathrm{DOF}\;(\sqrt{MSE})$	X (mm)	Y (mm)	Z (mm)	Pitch (°)	Yaw (°)	Roll (°)	Average
Training	-	-	-	-	-	-	0.071
Validation	-	-	-	-	-	-	0.13
Testing	0.14	0.14	0.15	0.12	0.10	0.12	0.13
Testing (unseen head model)	0.26	0.52	0.35	0.42	0.30	0.26	0.36

## Data Availability

The data presented are available on request from the corresponding author.
